# Identification of lncRNAs associated with T cells as potential biomarkers and therapeutic targets in lung adenocarcinoma

**DOI:** 10.32604/or.2023.042309

**Published:** 2023-09-15

**Authors:** LU SUN, HUAICHENG TAN, TING YU, RUICHAO LIANG

**Affiliations:** 1 Department of Targeting Therapy & Immunology, Cancer Center, West China Hospital, Sichuan University, Chengdu, 610041, China; 2 Department of Radiation Oncology, Cancer Center, West China Hospital, Sichuan University, Chengdu, 610041, China; 3 Department of Pathology, West China Hospital, Sichuan University, Chengdu, 610041, China; 4 Department of Neurosurgery, West China Hospital, Sichuan University, Chengdu, 610041, China

**Keywords:** Biomarkers, T cell-related lncRNAs, Tumor classification, Tumor treatment, Lung adenocarcinoma

## Abstract

Lung adenocarcinoma (LUAD) is the most common and deadliest subtype of lung cancer. To select more targeted and effective treatments for individuals, further advances in classifying LUAD are urgently needed. The number, type, and function of T cells in the tumor microenvironment (TME) determine the progression and treatment response of LUAD. Long noncoding RNAs (lncRNAs), may regulate T cell differentiation, development, and activation. Thus, our aim was to identify T cell-related lncRNAs (T cell-Lncs) in LUAD and to investigate whether T cell-Lncs could serve as potential stratifiers and therapeutic targets. Seven T cell-Lncs were identified to further establish the T cell-related lncRNA risk score (TRS) in LUAD. Low TRS individuals were characterized by robust immune status, fewer genomic alterations, and remarkably longer survival than high TRS individuals. The excellent accuracy of TRS in predicting overall survival (OS) was validated in the TCGA-LUAD training cohort and the GEO-LUAD validation cohort. Our data demonstrated the favorable predictive power of the TRS-based nomogram, which had important clinical significance in estimating the survival probability for individuals. In addition, individuals with low TRS could respond better to chemotherapy and immunotherapy than those with high TRS. LINC00525 was identified as a valuable study target, and the ability of LUAD to proliferate or invade was significantly attenuated by downregulation of LINC00525. In conclusion, the TRS established by T cell-Lncs could unambiguously classify LUAD patients, predict their prognosis and guide their management. Moreover, our identified T cell-Lncs could provide potential therapeutic targets for LUAD.

## Introduction

Lung cancer is the second most prevalent cancer and leading cancer killer worldwide [1]. Clinical data demonstrate that approximately 85% of all lung cancers are non-small cell lung cancer (NSCLC), with the most predominant type being lung adenocarcinoma (LUAD) [2,3]. LUAD progresses rapidly with micrometastatic foci and has a high recurrence rate and an increased metastatic rate. Standard surgery is performed in patients with locally confined and early-stage disease, but an overwhelming number of patients tend to have advanced disease, undergo traditional treatment such as combined radiotherapy and chemotherapy, and experience an elevated mortality risk [4]. However, the paradigm of LUAD treatment has been altered dramatically in the last two decades. Molecular targeted therapies and immunotherapies have markedly improved outcomes for many patients. This is largely due to the availability of biomarkers that can be exploited to screen candidates receiving targeted therapy or immunotherapy [5]. Despite these advances in targeted and immune-based therapies, the overall survival (OS) of 5 years for LUAD patients is unfortunately still less than 20% [2]. The development of reliable predictive biomarkers is still essential for the unambiguous stratification of patients, which can provide clues to prognosis, guide treatment decisions, and optimize therapeutic effects for LUAD individuals.

T cells are considered the most important driving force in tumor immunity. Many studies have reported that the infiltration of T cells into the tumor microenvironment (TME) correlates with favorable outcomes of several malignancies in humans [6,7]. For example, in patients with resected NSCLC, the extent of T cell infiltrates in the central cancer stroma has been demonstrated to be a favorable prognostic biomarker [8]. Intratumoral T cells are generally divided into CD8^+^ and CD4^+^ T cell subtypes. While CD8^+^ T cells mainly exert antitumor immunity, CD4^+^ T cells exert dual functions in tumor control by performing either protumor or antitumor functions [9]. Regulatory T cells (Tregs), a highly immunosuppressive subtype of CD4^+^ T cells, have been demonstrated to counteract effective antitumor immunity by impeding the infiltration and antitumor responses of CD8^+^ T cells and macrophages [10]. However, recent technological advances in single-cell analyses of transcriptional and phenotypic states have highlighted that the functional states of intratumoral T cells are highly heterogeneous both intra- and inter-patient [11]. T cell exhaustion is a term used to describe the alteration of the PD-1^+^ T cell functional state, and the proportion of pre-exhausted T cells to exhausted T cells is linked to improved outcomes in LUAD patients [12,13]. It appears that the number, type, and functional state of infiltrating T cells are closely related to tumor prognosis. Therefore, additional studies should further focus on the biomarkers implicated in regulating intratumoral T cell infiltration, differentiation, and function to better predict the prognosis for LUAD individuals.

Long noncoding RNAs (lncRNAs) are a subset of noncoding RNA molecules. Their noncoding transcripts are longer than 200 nucleotides [14]. Cancer transcriptome analyses have revealed that several thousand lncRNAs are linked to multiple types of cancer [15]. LncRNAs within body fluids are able to mimic aberrant profiles of lncRNAs within the TME [16]. For example, overexpression of five specific lncRNAs within the TME correlates with worse gastric cancer (GC) outcomes, and tracking the aberrant expression of five specific lncRNAs within body fluids can effectively discriminate between GC patients and healthy individuals, as well as various stages of GC patients [17]. LncRNAs also actively participate in immune regulation in 33 types of cancer [18]. During immune response activation, especially T cell differentiation, development and activation, extensive changes in lncRNA expression occur [19]. Twenty-eight common lncRNAs have been proven to regulate immune-related signaling pathways in lung cancer, with dramatically increased expression in T and B cells [18]. Moreover, lncRNAs have been demonstrated to play a crucial role in immune escape by altering the balance between immunoreactive and immunosuppressed T cells [20,21]. LncRNA-epidermal growth factor receptor (EGFR), which is upregulated in Tregs, promotes immune evasion of hepatocellular carcinoma by stimulating Treg differentiation and suppressing cytotoxic T lymphocyte (CTL) activation [22]. NF-κB-interacting lncRNA (NKILA), which is overexpressed in CTLs, mediates CTL apoptosis through activation-induced cell death (AICD) in breast cancer and lung cancer [23]. Considering that lncRNAs are easily detectable and have implications in T cell regulation, a comprehensive study of the complex interactions of T cell-related lncRNAs (T cell-Lncs) in the TME is interesting and valuable. However, little is known about the T cell-Lncs involved in LUAD.

In this study, seven T cell-Lncs were identified to develop the T cell-related lncRNA risk score (TRS) for LUAD patients. On the basis of median TRS, patients suffering from LUAD were classified into two subgroups, which had different survival prospects, mutational characteristics, and immune contexture in their TME. Moreover, TRS was highly accurate at predicting survival and treatment response for LUAD individuals. LINC00525, as the only risk factor among the seven T cell-Lncs, could help LUAD proliferate and invade.

## Materials and Methods

### Data source and processing

**Figure S1:**
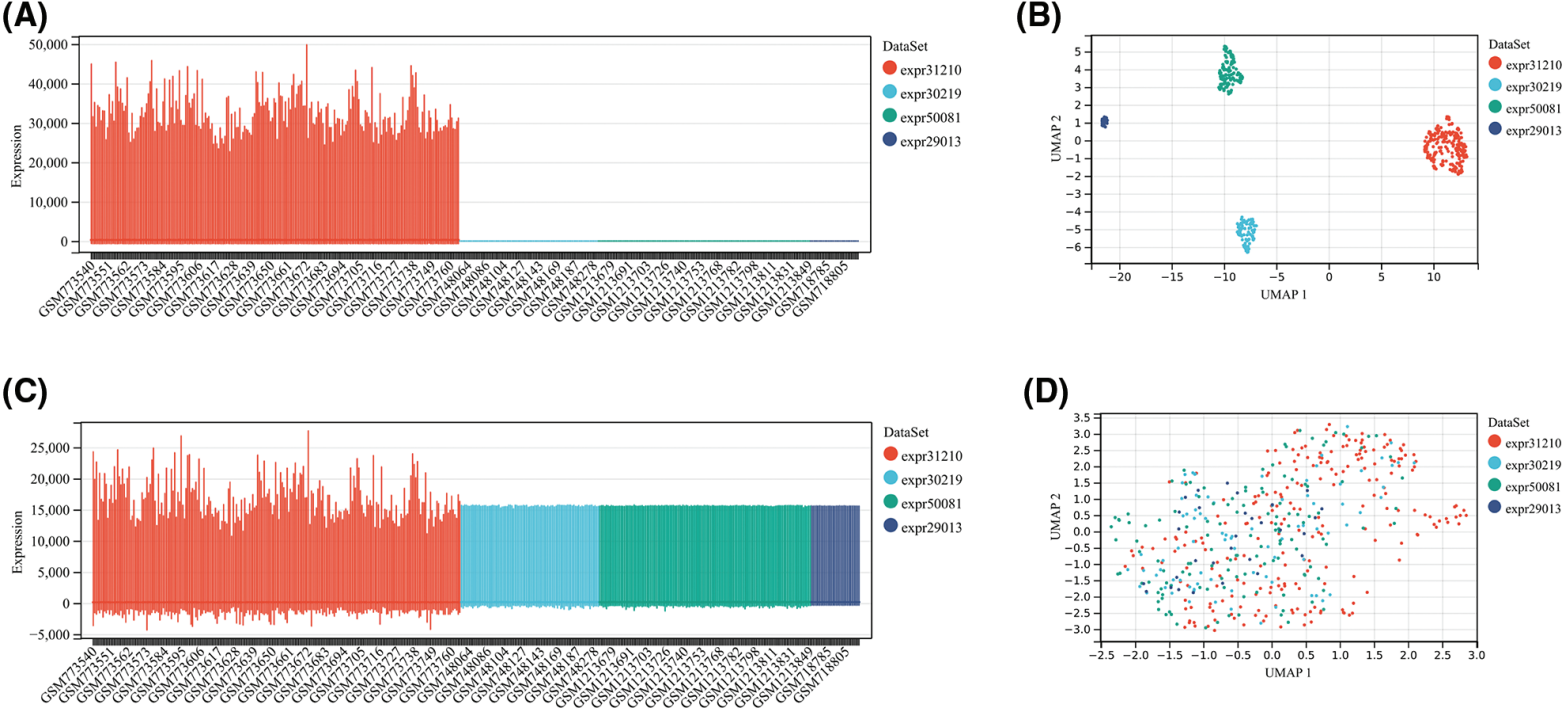
The evaluation of pre-integrate and post-integrate data distribution with Boxplot and umap. Data distribution before integration of four datasets including GSE31210, GSE30219, GSE50081, and GSE29013 evaluated by Boxplot (A) or umap (B). Data distribution after integration of four datasets including GSE31210, GSE30219, GSE50081, and GSE29013 evaluated by Boxplot (C) or umap (D).

Gene expression and clinical data of all available LUAD patients were collected using the The Cancer Genome Atlas (TCGA) and Gene Expression Omnibus (GEO) databases. Fragment per kilobase million (FPKM) values were normalized to transcript per kilobase million (TPM) values to perform the following analyses. The TCGA-LUAD cohort with 492 patients was utilized as the training cohort. From the GEO database, we collected four datasets, including GSE29013, GSE30219, GSE31210, and GSE50081. The package “SVA” was employed to run the algorithm “ComBat” to integrate these four datasets, remove batch effects and normalize. Then, the GEO dataset, containing 461 LUAD patients, was used as an external validation cohort. Boxplot and umap were performed to assess the pre-integrate and post-integrate data distribution, and these obtained results showed that batch effects were well removed (Suppl. Fig. S1). We obtained the IMvigor210 dataset from a data package that was freely available. The IMvigor210 dataset, including 298 urothelial cancer patients undergoing immunotherapy, was utilized to examine the predictive value of TRS. Finally, we collected transcriptional profiles of the TCGA-pancancer cohort to assess the predictive efficacy of TRS in the pancancer landscape.

### Identification of T cell-Lncs in LUAD by two algorithms

The R package “ConsensusClusterPlus” was applied to implement consensus clustering in the training cohort [24]. The method used was a k-means algorithm based on Euclidean distance with 1,000 iterations and 80% sample selection for each iteration. The number of clusters was in the range of 2 to 5, and the proportion of ambiguous clusters (PAC) was exploited to acquire the optimal number. The module eigengenes (ME) closely associated with clinical traits (stage and clusters) were recognized through weighted gene coexpression network analysis (WGCNA) utilizing the R package [25]. We used the following parameters to build the “signed” scale-free network: “cutR = 0.9”, “cutNet = 0.02”, “deepSplit = 2”, “mergeCutHeight = 0.4”, and “minModuleSize = 30”. Based on the ImmLnc algorithm, the same pipeline was used for the calculation of lncRNAs associated with T cells [18].

### Establishment of the TRS model

To determine the best prognostic model, the Cox proportional hazards model with a least absolute shrinkage and selection operator (LASSO) penalty was utilized, and the model was subjected to fivefold cross-validation to ensure its stability. Considering random sampling, we performed 300 iterations to confirm the most stable prognostic model. Finally, seven genes were proposed to establish the TRS. By using the following formula, TRS was calculated for each of the patients. Finally, the LUAD patients were stratified by the median value of TRS.

$$TRS=\sum iCoefficient\left(mRNA_{i}\right)\times Expression\left(mRNA_{i}\right)$$

### Assessment of TRS as a prognostic model in LUAD

The prognostic value of TRS and three clinical indicators was statistically evaluated and contrasted by using the “survcomp” package. We estimated the concordance index (C-index) for each indicator separately across the TCGA-LUAD training and GEO-LUAD validation cohorts and applied the combine.est function for calculating the respective total meta-estimate [26]. A higher C-index indicated that the risk prediction model was more accurate. The prognostic independence of TRS in different LUAD cohorts was evaluated with both multivariate and univariate Cox regression analyses. In addition, time-dependent receiver operating characteristic (ROC) curves of the TRS were plotted to assess the predictive ability at different survival times.

### Building and evaluation of a nomogram

For evaluating survival probability in 1-year (1y), 3-year (3y), and 5-year (5y) of LUAD individuals, the prognostic nomogram was established with the rms R package. Gender, stage, age, and TRS were used as independent parameters. Calibration curves were plotted to compute discrimination and calibration between actual and nomogram-predicted values. To quantify the net benefit at different threshold probabilities, we employed decision curve analysis (DCA), which has been routinely applied to evaluate the clinical availability of alternative models.

### Functional enrichment analysis and collection of immune-related data

The differentially expressed genes (DEGs) of the two subgroups were recognized utilizing the “limma” package, with the screening thresholds set to |log_2_ fold change (FC)| > 1 and false discovery rate (FDR) < 0.05. Upregulated DEGs from both subgroups were uploaded to Metascape (https://metascape.org/) for functional enrichment analyses. The different bioprocesses in each subgroup were investigated by gene set enrichment analysis (GSEA) utilizing the “GSVA” R package. We conducted Kyoto Encyclopedia of Genes and Genomes (KEGG) pathway enrichment analyses in the two subgroups, and significantly enriched the pathway with FDR < 0.05 [27]. We further acquired the 50 hallmark gene sets from the Molecular Signatures Database (MSigDB). To evaluate the activity of the hallmark pathways and the Pearson correlation of TRS with the hallmark pathways, single-sample GSEA (ssGSEA) was utilized [28]. Proliferation, tumor mutational burden (TMB), intratumor heterogeneity, leukocyte fraction, lymphocyte infiltration signature, transforming growth factor beta (TGF-beta) response, tumor-infiltrating lymphocyte (TIL) regional fraction, insertion/deletion (Indel) neoantigens, single nucleotide variant (SNV) neoantigens, microsatellite instability (MSI), and homologous recombination deficiency (HRD) scores were examined in accordance with the report of Thorsson et al. [29]. We exploited the ESTIMATE algorithm for the calculation of immune, stromal, and ESTIMATE scores to further estimate tumor purity. The “CIBERSORT” R package was employed for the evaluation of immune cell infiltrates in the samples. In addition, we conducted ssGSEA to examine the samples' immune-related pathway activity based on previously reported gene markers [30]. Specific gene markers of each pathway are available in Suppl. Table S1.

### Analysis of genomic alterations in both subgroups

We extracted mutational signatures from the whole-exome sequencing (WES) data with the “Sigminer” R package [31]. To decode somatic mutation signatures in cancer, we implemented a Bayesian variant of the non-negative matrix factorization (NMF) algorithm based on 96 substitutions in the context of trinucleotide sequences. When the NMF algorithm determined the optimum k-factorization value (k = 4), four mutational signatures were found in both subgroups. An individual score was generated for each mutational signature by the non-negatively constrained least squares (NNLS) algorithm, which combined probability with cosine similarity and signature exposure. Annotation of mutational signatures was performed by calculating cosine similarity using recognized mutation catalogs for single base substitutions (SBS) from the COSMIC database [32,33]. The R package “maftool” was conducted to analyze mutation load in the two subgroups [34]. The OncodriveCLUST algorithm was used to identify mutant genes in cancer based on protein fragments with significant aggregation of gain-of-function mutations, defining the top 20 mutant genes as driver mutant genes. The copy number alteration (CNA) data were processed using Gistic2.0 software. Then, the number of amplified or deleted chromosomal segments was determined based on a threshold value of 0.2. Finally, the package “RCircos” was employed to visualize and display these CNA results from LUAD patients. The mutational landscape of LUAD patients across distinct TRS groups is shown by a complex heatmap.

### Prediction of therapeutic benefits based on TRS

The R package “pRRophetic” was employed for sensitivity analysis of pharmacological agents, including vinorelbine, cisplatin, gemcitabine, docetaxel, and paclitaxel, according to the Genomics of Drug Sensitivity in Cancer (GDSC). We performed ridge regression for the calculation of the half-maximal inhibitory concentration (IC50) estimate for each sample, and tenfold cross-validation was performed to ensure predictive accuracy [35,36]. The Connectivity Map (CMap) database enabled the establishment of connections between biologically active small molecules and gene expression that could predict small molecule agents for the treatment of specific diseases on the basis of gene expression characteristics [37]. DEGs between the two subgroups were entered into the CMap database for the prediction of potential small molecule drugs for LUAD treatment. In addition, −90 was selected as the prediction threshold to identify drug candidates for LUAD treatment. Meanwhile, we conducted CMap mode-of-action (MOA) analyses to identify the underlying mechanisms by which a drug acted [36]. We calculated the immunophenoscore (IPS) of the two subgroups for the prediction of response to programmed cell death 1 (PD-1) and cytotoxic T lymphocyte-associated protein-4 (CTLA-4) blockade immunotherapy in LUAD patients. According to the transcriptome of the genes that were representative of the immunophenotype, the IPS was determined on a 0 to 10 scale. Samplewise Z scores were weighted positively based on effective immune cells and negatively based on inhibitory immune cells, and then the mean values were taken. Z score ≤ 0 was referred to as IPS0, and Z score ≥ 3 was referred to as IPS10 [38]. We also evaluated the sensitivity of anti-CTLA-4 and anti-PD-1 drugs to LUAD patients from two subgroups using the Tumor Immune Dysfunction and Exclusion (TIDE) algorithm and subclass mapping algorithm.

### Evaluation of the impact of LINC00525 on LUAD cell proliferation or invasion

The human lung adenocarcinoma cell strain (A549 cell) or the human bronchial epithelial cell strain (16HBE cell) was acquired from ATCC. A549 cells or 16HBE cells were incubated in DMEM (Gibco, USA). We transfected A549 cells with small interfering RNAs (siRNAs) utilizing Lipofectamine 3000 transfection kits, as described in the manufacturer’s protocol. The designed and synthesized siRNA LINC00525 (siLINC00525) and siRNA negative control (siNC) were competed by GeneChem. We used the following siRNA sequences: si-LINC00525: 5′-UAAAAUCGGAAUUCCUUUCAC-3′, si-NC: 5′-GAAAGGAAUUCCGAUUUUAAA-3′. Quantitative Real Time PCR (qRTPCR) analyses were carried out to determine the expression of LINC00525 in 16HBE cells, A549 cells, and A549 cells that were transfected with siNC or siLINC00525. Total cellular RNA was obtained using TRIzol reagent (Invitrogen, USA). Following cDNA synthesis (TAKARA, China), SYBR green supermix (Bio-Rad, USA) was used to perform qRTPCR assays. The primer sequences were as follows: GAPDH, forward, 5′-CACCATCTTCCAGGAGCGAG-3′, reverse, 5′-CTTCTCCATGGTGGTGAAGAC-3′; LINC00525, forward, 5′-TGCAACTACGACCCCGAAAA-3′, reverse, 5′- GTGGATGTACGGTGCAAGGA-3′. The CCK-8 assay was run by using the CCK-8 kit according to the manufacturer’s instructions (CK04-500T). Absorbance at 450 nm was monitored with the Thermo Fisher Scientific Multiskan Go. For the Transwell assay, 5 × 10^4^ A549 cells transfected with siNC or siLINC00525 in serum-free medium were placed in the top chambers of BioCoat Matrigel invasion chambers (354480). The bottom chambers contained DMEM with 10% FBS. Invasive cells were fixed, stained and counted after incubation for 48 h.

### Statistical analyses

R software (v. of 4.0.4) was applied to carry out all statistics in this study. Between-group differences were evaluated by *t* test or Wilcoxon test. Survival analysis was performed on each dataset by means of the Kaplan‒Meier plotter, and the log-rank test was exploited to determine significant differences. The chi-square test was run to analyze correlations between categorical variables. Pearson analysis was performed to compute correlation coefficients. A two-tailed *p* < 0.05 was deemed to have statistical significance except as noted above.

## Results

### Identification of T cell-Lncs by two algorithms in LUAD

**Figure 1:**
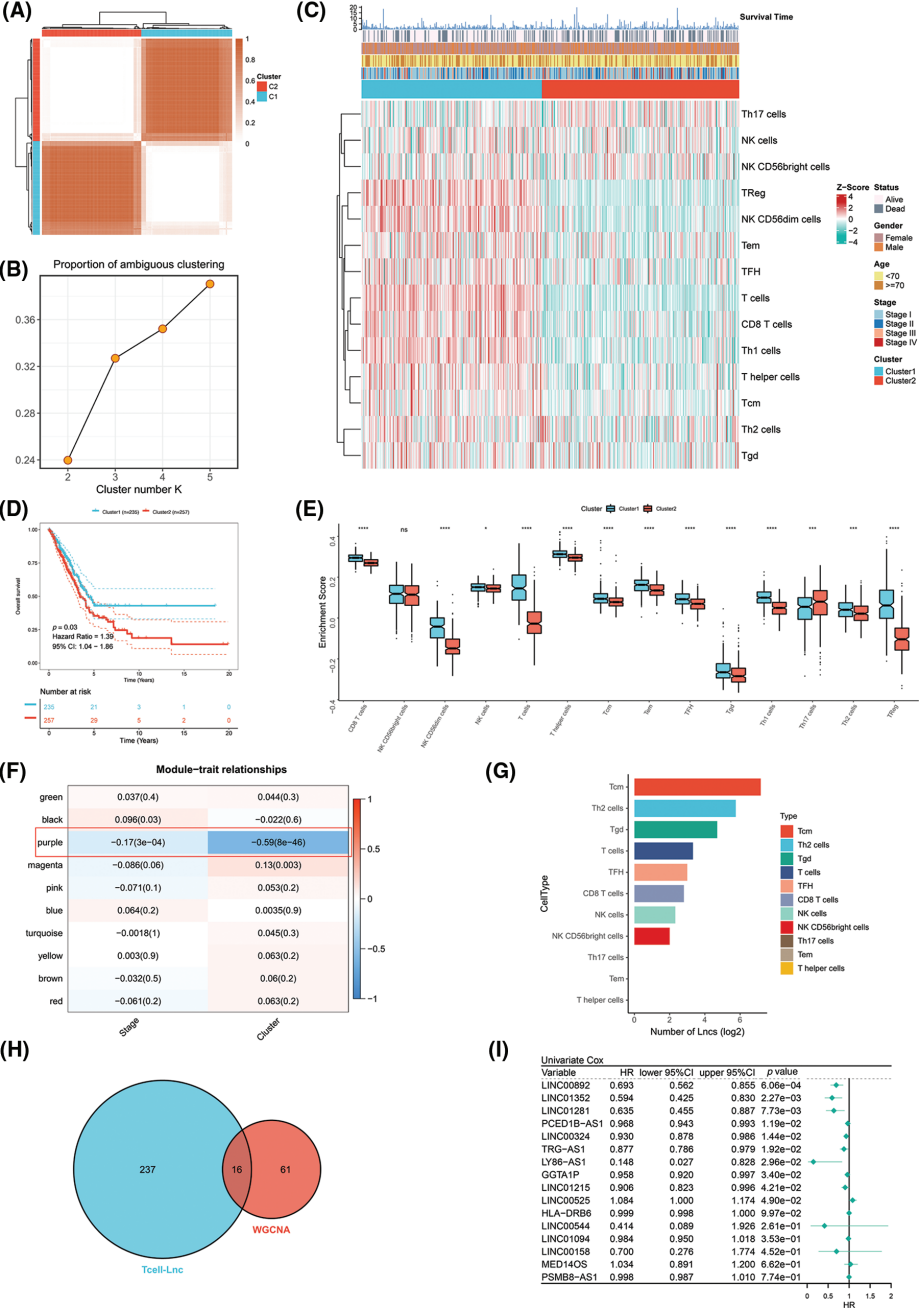
Identification of T cell-Lncs in LUAD by two algorithms. (A) The consensus score matrix of all samples when k = 2 in the TCGA-LUAD training cohort. (B) The PAC score; a low value of PAC indicated an apartment middle segment and allowed the conjecture of the optimum k (k = 2) by the lowest PAC. (C) The heatmap shows the clinicopathologic features and immune cell infiltration of the two clusters. (D) Survival analysis of TCGA-LUAD patients in the two clusters. (E) Enrichment analysis of immune cells in the two clusters (ns, not significant; *p < 0.05; **p < 0.01; ***p < 0.001; and ****p < 0.0001). (F) Heatmap of the correlations between ME and clinical traits (stage or cluster). Each row represents one ME, and each column represents one trait. Each cell contained the specific correlation index and *p* value. (G) The number of lncRNAs (Lncs) correlated with each immune cell type. (H) The intersection between the ImmLnc algorithm (blue) and the WGCNA algorithm (red). (I) Univariate Cox regression analysis of the 16 T cell-Lncs.

The TCGA-LUAD patients were specified as the training cohort and clustered into k groups (k = 2–5) using the “ConsensusClusterPlus” R package. We found it optimal to divide the cohort into two clusters. The PAC method also proved the optimum number for k = 2 (Figs. 1A and 1B). We then examined the clinicopathologic features and infiltration by immune cells in the two clusters. A marked difference in the extent of T cell infiltrates was observed in the two clusters (Fig. 1C). Survival analysis revealed that Cluster 1 (C1) achieved a more favorable OS than Cluster 2 (C2) in the TCGA-LUAD cohort (Fig. 1D). As a further analysis, enrichment of immune cells within both clusters was examined by ssGSEA. C1 and C2 showed a significant difference in immune cell enrichment, with the exception of NK CD56bright cells. C1 showed markedly increased enrichment of T cells except Th17 cells (Fig. 1E). On the basis of WGCNA, the correlation analysis of ME with clinical traits (stage or cluster) revealed several findings. The purple module, unlike the other modules, was most negatively correlated with cluster (R = −0.59, *p* = 8E-46) and stage (R = −0.17, *p* = 3E-04). We also preliminarily filtered out a set of 77 candidate genes in the purple module for subsequent analysis (Fig. 1F). LncRNAs are known to have important roles in regulating the expression of immune system genes. In particular, lncRNAs linked to immunity might be aberrantly expressed within cancer and strikingly associated with immune cell infiltration [18]. Considering that both clusters differed in the infiltration level of immune cells, especially T cells, we used an integrated algorithm, ImmLnc, to determine the lncRNAs linked to each immune cell type within LUAD. A total of 253 genes most relevant to T cells were found and identified as T cell-Lncs, as shown in Fig. 1G. We then took the intersection between 253 genes related to T cells identified by the ImmLnc algorithm and 77 genes related to clusters identified by the WGCNA algorithm. A total of 16 T cell-Lncs were in the intersection of the two algorithms and were selected as candidate T cell-Lncs (Fig. 1H). Subsequently, 16 T cell-Lncs were evaluated utilizing univariate COX regression analysis, and 10 T cell-Lncs with independent prognostic efficacy were screened out for further analysis (Fig. 1I).

### Construction of the TRS model

**Figure 2:**
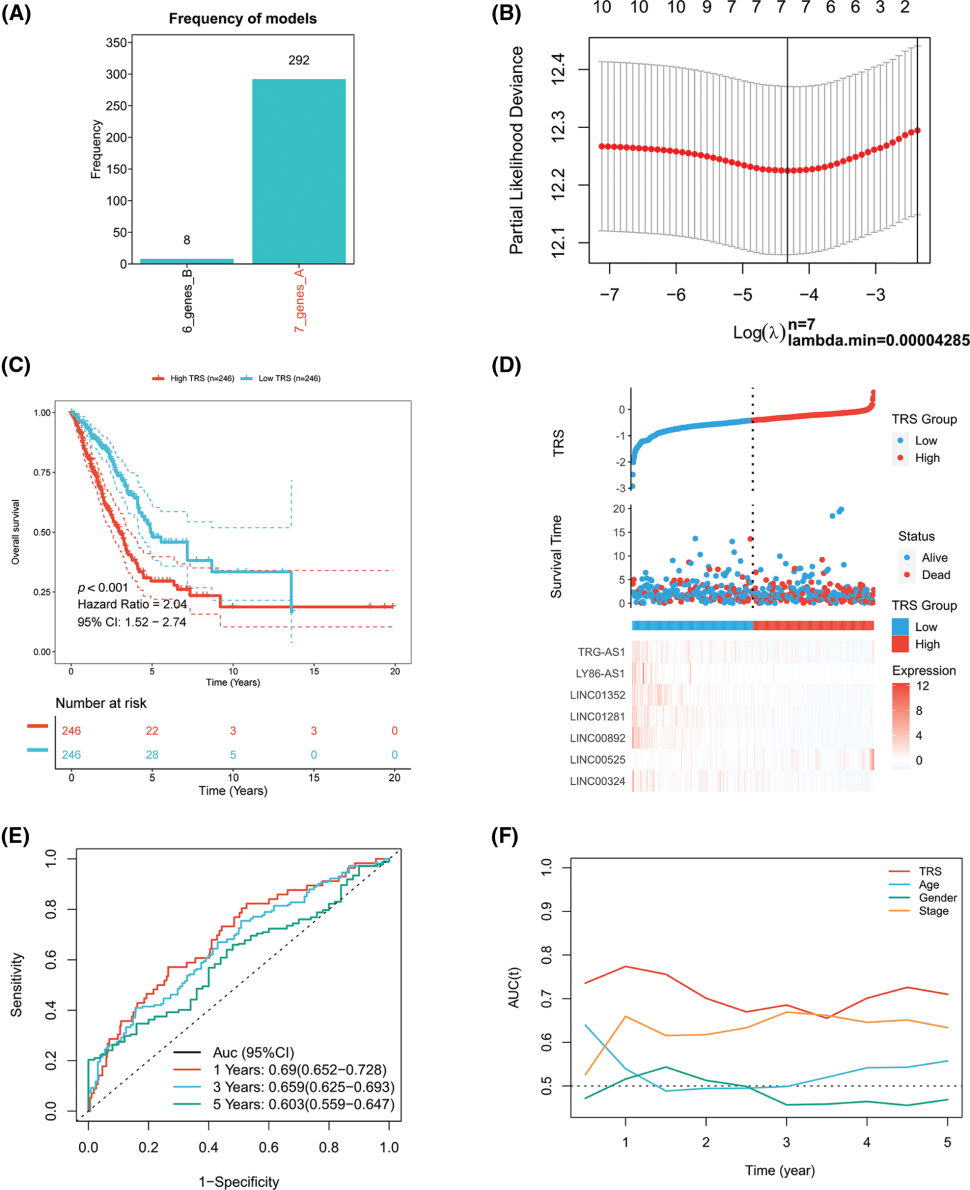
Construction of the TRS model. (A) Frequency of models in LASSO-Cox analysis. (B) Partial likelihood deviation of LASSO coefficient distribution. (C) Kaplan‒Meier analysis of LUAD patients with high and low TRS from the TCGA-LUAD training cohort. (D) The distributions of TRS and survival status and the expression distributions of seven T cell-Lncs in the TCGA-LUAD training cohort. (E) ROC curves for 1 years, 3 years, and 5 years survival times based on TRS in the TCGA-LUAD training cohort. (F) The time-dependent AUC values of TRS, age, gender, and stage for OS prediction in the TCGA-LUAD training cohort.

**Figure S2:**
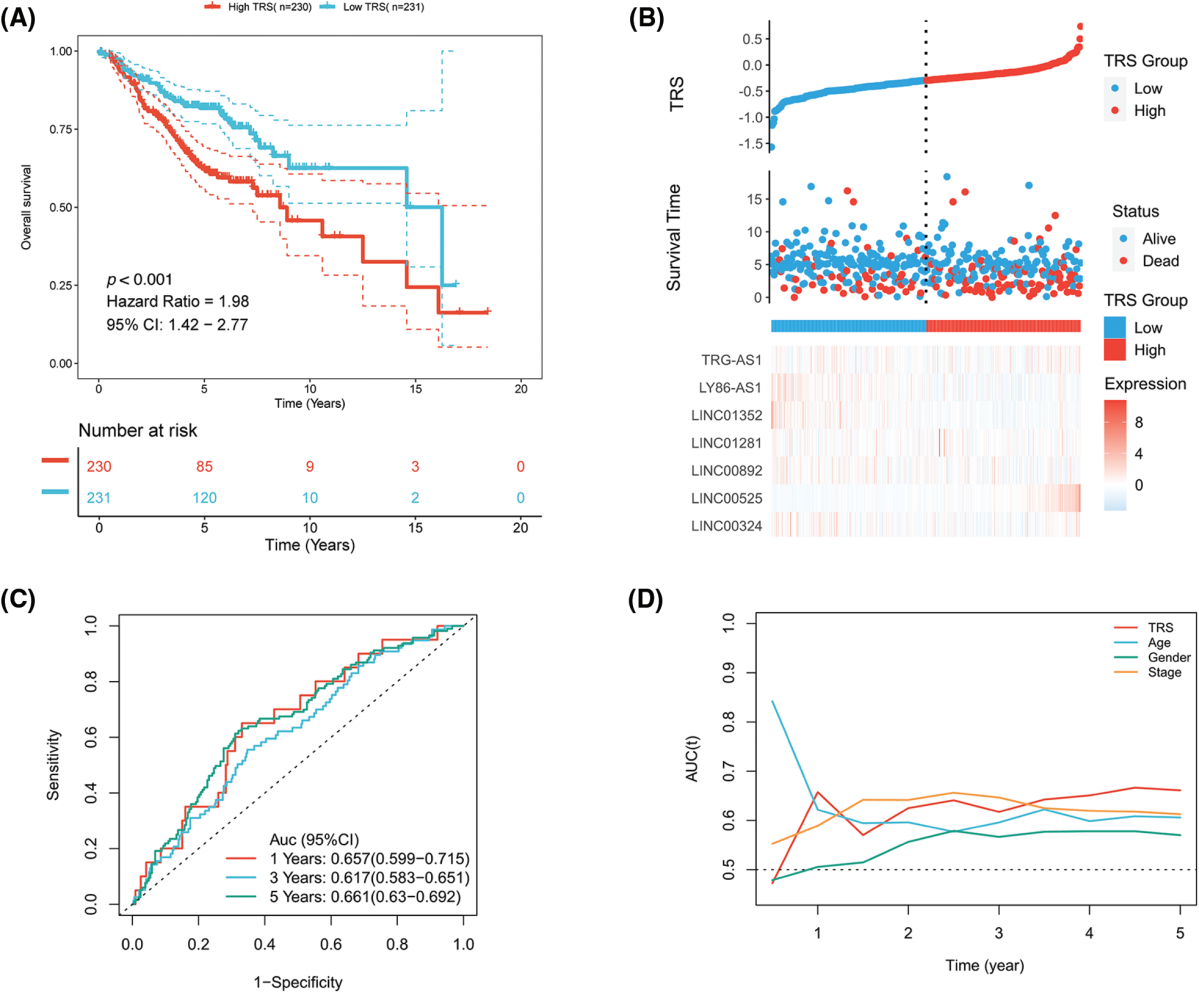
The evaluation of TRS as a prognostic model in the GEO-LUAD validation cohort. (A) Kaplan-Meier analysis of LUAD patients with high and low TRS from the GEO-LUAD validation cohort. (B) The distributions of TRS and survival status and the expression distributions of seven T cell-Lncs in the GEO-LUAD validation cohort. (C) ROC curves for 1 years, 3 years, and 5 years survival times based on TRS in the GEO-LUAD validation cohort. (D) The time-dependent AUC values of TRS, age, gender, and stage for OS prediction in the GEO-LUAD validation cohort.

A Cox proportional hazards model with a lasso penalty was exploited to develop a model for the TRS. A set of seven genes emerged as the most stable prognostic model with 300 iterations performed in the LASSO-Cox analysis, as illustrated in Fig. 2A. The seven lncRNAs LINC00324, LINC00892, LINC01281, LINC01352, LY86-AS1, TRG-AS1, and LINC00525 then formed the most stable model for TRS based on the minimum lambda = 0.00004285 of the LASSO algorithm (Fig. 2B). Each lncRNAs risk coefficient is presented in Suppl. Table S2. We sought to further determine the TRS’s utility in predicting patient outcomes. LUAD patients were stratified as high TRS/low TRS depending on median TRS. In both the training TCGA-LUAD and validation GEO-LUAD cohorts, low TRS individuals experienced a statistically significant survival benefit compared to high TRS individuals (Fig. 2C and Suppl. Fig. S2A). The distributions of TRS and survival status, as well as the expression distributions of seven T cell-Lncs, were also analyzed in two cohorts (TCGA-LUAD training, GEO-LUAD validation) (Fig. 2D and Suppl. Fig. S2B). As the TRS increased, those with high TRS did not have a better prognosis than those with low TRS. Six of seven T cell-Lncs, including TRG-AS1, LY86-AS1, LINC01352, LINC01281, LINC00892, and LINC00324, were clearly elevated in low TRS individuals, while only a single T cell-Lnc, LINC00525, was significantly upregulated in high TRS individuals. To assess the predictability when using the TRS in TCGA-LUAD patients, we created an ROC curve. For 1-year, 3-year, and 5-year survival times, the area under the curve (AUC) values obtained were 0.69, 0.659, and 0.603, respectively. These data revealed that TRS predicted survival in LUAD patients with high accuracy (Fig. 2E). Based on the time-dependent AUC values, TRS had superior predictive power for OS at any time point in five years compared with age, gender, and stage (Fig. 2F). Additionally, the GEO-LUAD cohort, which showed similar results, also validated the value of TRS as a prognostic tool (Suppl. Figs. S2C and S2D).

### Evaluation of TRS as a prognostic tool and construction of a TRS-based nomogram in LUAD

**Figure 3:**
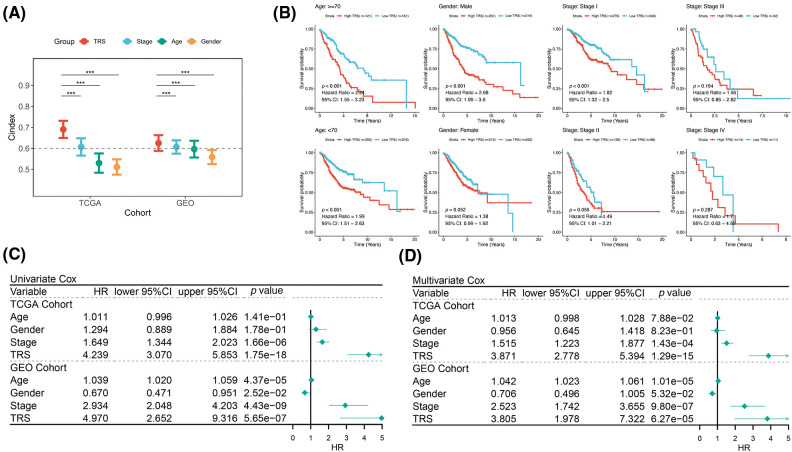
Evaluation of TRS as a prognostic model in LUAD. (A) The prognostic value of TRS, stage, age, and gender were estimated by the C-index in both the TCGA-LUAD training cohort and GEO-LUAD validation cohort. (B) Survival analysis of TRS in different clinical subgroups. (C) Univariate Cox regression analysis of age, gender, stage, and TRS in both the TCGA-LUAD training cohort and GEO-LUAD validation cohort. (D) Multivariate Cox regression analysis of age, gender, stage, and TRS in both the TCGA-LUAD training cohort and GEO-LUAD validation cohort.

The prognostic value of TRS has been studied in more detail. In each cohort, the C-index of TRS and three clinical indicators, including stage, age and gender, were calculated. As illustrated by Fig. 3A, TRS possessed a higher C-index in both cohorts when compared to each of the three clinical indicators. This result was an indication that TRS has a high degree of accuracy in predicting LUAD patient outcomes. Meanwhile, to investigate the prognostic value of TRS across different clinical subgroups, we integrated the TCGA-LUAD training and GEO-LUAD validation cohorts. When we stratified patients with LUAD by gender, age, and stage of disease, those with high TRS tended to experience worse outcomes (Fig. 3B). Notably, survival outcomes for early-stage LUAD (stages I and II) differed significantly across high- and low-TRS arms in this study. This suggests that TRS may be more effective in predicting prognosis in LUAD patients with early-stage disease. Univariate Cox regression analysis demonstrated that TRS was an objective prognostic factor in LUAD cases from the TCGA- and GEO-LUAD cohorts (Fig. 3C). Further evidence was provided by multivariate Cox regression analysis. TRS continued to be a reliable prognostic model after adjustment for other clinical factors (Fig. 3D).

**Figure 4:**
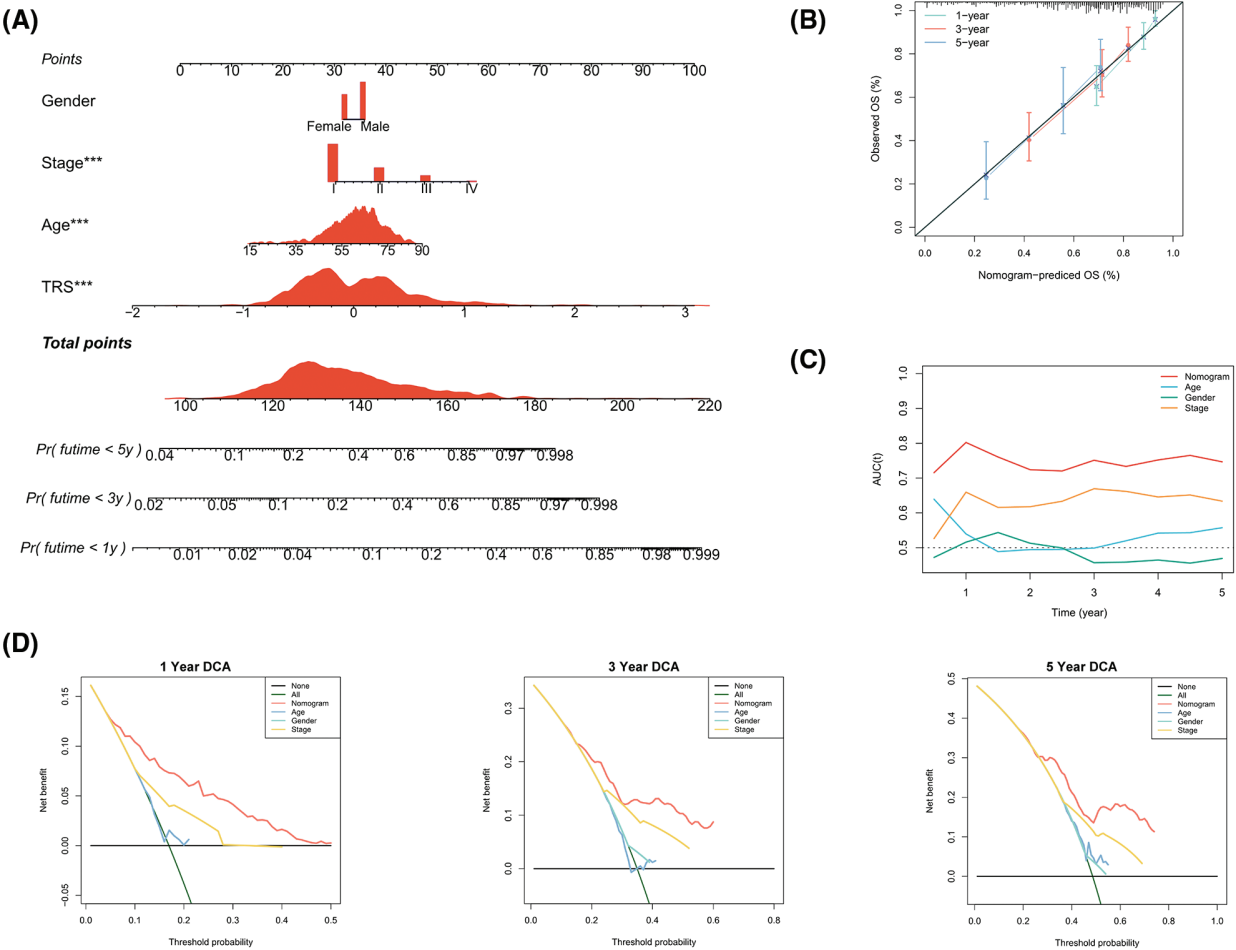
Construction of a TRS-based nomogram to quantify risk for individual patients. (A) A nomogram integrating TRS, gender, stage, and age was constructed to predict 1-year, 3-year, and 5-year survival rates for individual patients. To compute the probability of survival, determine the patient values on each axis and then draw a vertical line upward to the point axis for each. Add up the points for all variables and then put this sum on the line for the total points (*p < 0.05, **p < 0.01, and ***p < 0.001). (B) Calibration curves of the nomogram in terms of agreement between predicted and observed 1-year, 3-year, and 5-year OS. (C) The time-dependent AUC values of TRS, age, gender, and stage for OS prediction. (D) DCA at 1 year, 3 year, and 5 year for the nomogram, age, gender, and stage.

In view of the significant predictive value of TRS for patients with LUAD, our next step was to explore the clinical utility of TRS. We created a nomogram integrating TRS and multiple risk factors (gender, stage, and age) to assess the probability of survival for individual patients. The scores of the factors indicated their respective contribution to the probability of survival. Scores were summed to provide estimated 1-year (1y), 3-year (3y), and 5-year (5y) survival rates for each individual (Fig. 4A). As shown by the following calibration curves, the actual OS and the OS predicted by the nomogram agreed well at 1-year, 3-year, and 5-year (Fig. 4B). The results showed that the nomogram based on TRS was a remarkably stable model. Moreover, the time-dependent AUC values confirmed the better predictive performance of the TRS-based nomogram relative to age, gender, and stage (Fig. 4C). The TRS-based nomogram had the best decision net benefit at nearly all 1 year, 3 year, and 5 year threshold probabilities from a DCA perspective (Fig. 4D). In summary, the TRS-based nomogram could be used as an individualized quantitative tool to predict outcomes in LUAD.

### Analysis of TRS functional enrichment

**Figure 5:**
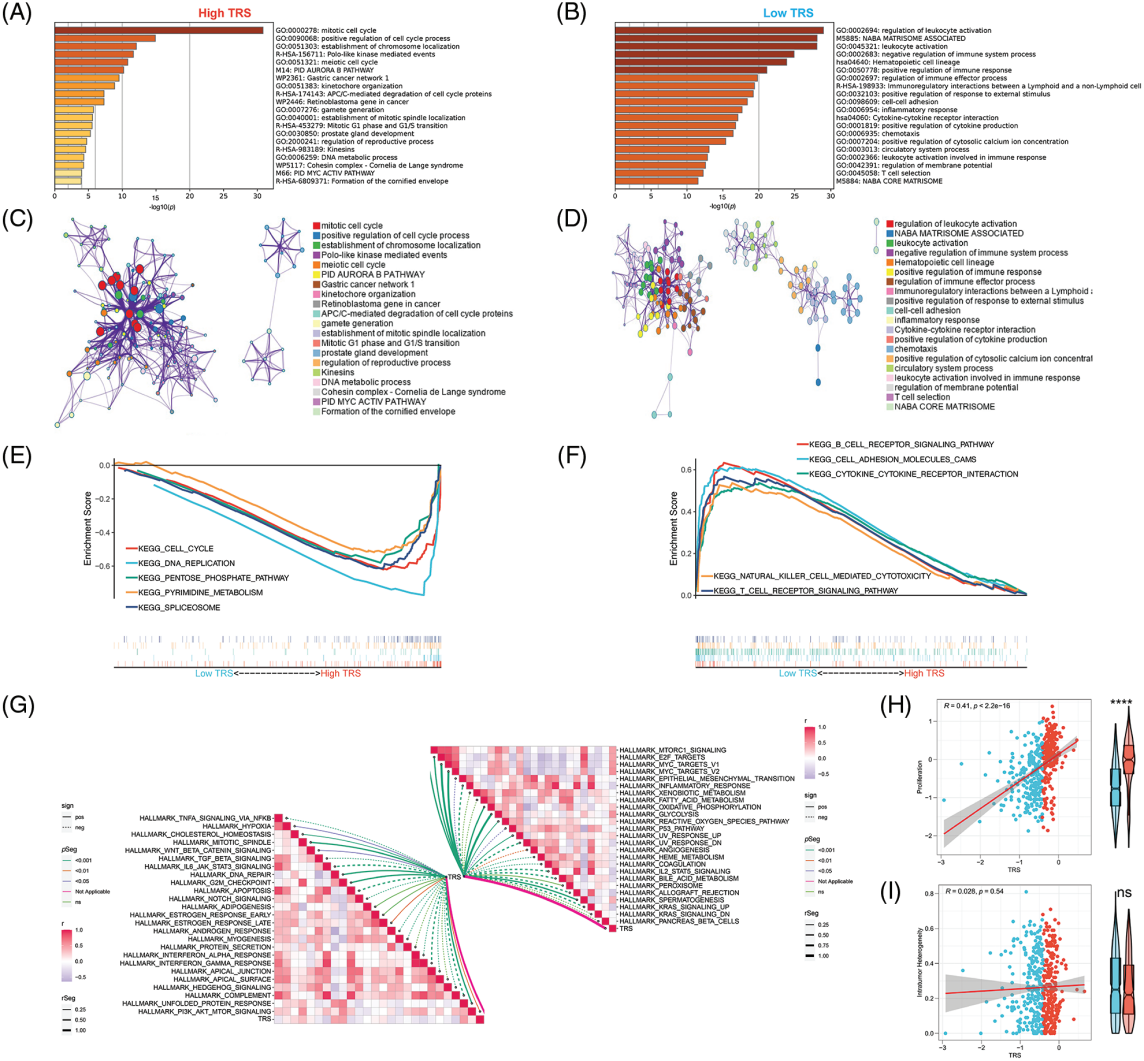
Functional enrichment analysis of TRS. Metascape enrichment analysis of the upregulated DEGs in the high (A) and low (B) TRS groups. Metascape enrichment network of the upregulated DEGs in the high (C) and low (D) TRS groups. KEGG pathway enrichment analyses of high (E) and low (F) TRS groups. (G) ssGSEA showed the correlation between TRS and 50 hallmarks. (H) Correlation analysis between TRS and proliferation. (I) Correlation analysis between TRS and intratumor heterogeneity.

In addition, functional enrichment analyses were performed to illustrate the bioinformatics functions of DEGs derived from different TRS groups. The DEGs that were upregulated in individuals with high TRS were found to be concentrated in signaling pathways linked to the cell cycle (Fig. 5A). In contrast, the DEGs, which were upregulated in individuals with low TRS, accumulated mainly in signaling pathways linked to the immune response (Fig. 5B). To further investigate the relationship between the enriched terms, we plotted them in a network diagram (Figs. 5C and 5D). Analyses of the enrichment of KEGG pathways also demonstrated that the signaling pathways correlated with the cell cycle were substantially enriched in high TRS individuals, whereas the signaling pathways associated with the immune response were predominantly enriched in low TRS individuals (Figs. 5E and 5F). In addition, TRS was found to positively correlate with hallmarks involved in proliferation. However, there was a negative correlation between TRS and hallmarks implicated in the immune response (Fig. 5G). Then, we also performed correlation analysis between TRS and proliferation. TRS appeared to have a positive and statistically significant relationship with proliferation (Fig. 5H) (Pearson coefficient R = 0.41, *p* < 2.2e-16). The individuals with high TRS had slightly higher proliferation than individuals with low TRS. However, TRS did not correlate with tumor heterogeneity (Pearson coefficient R = 0.028, *p* = 0.54, Fig. 5I). Taken together, these results suggested that the high TRS group was mainly relevant to more active cell proliferation, while the low TRS group was mainly relevant to a more active immune landscape.

### Analysis of the correlation between TRS and TME

**Figure 6:**
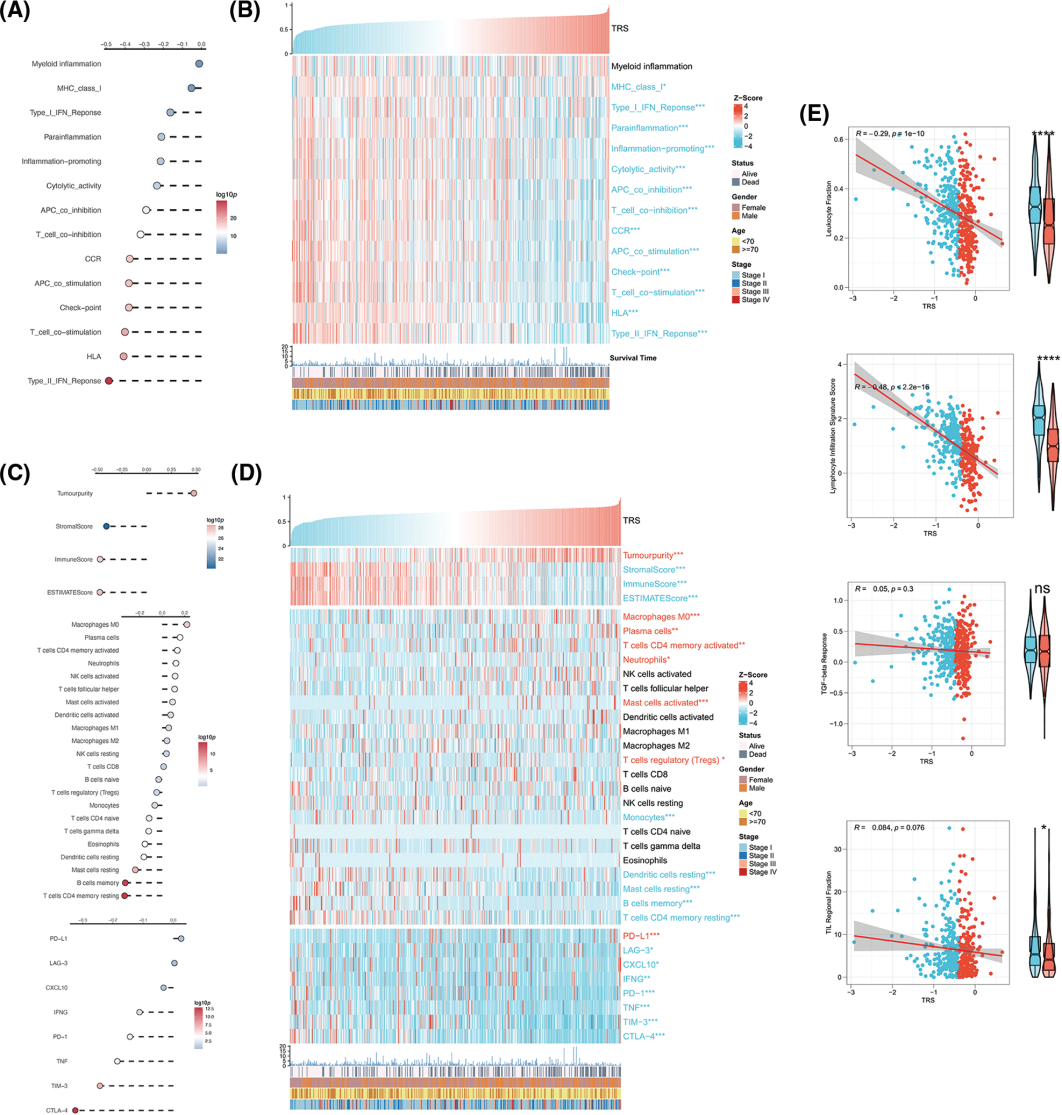
Correlation analysis between TRS and TME. (A) Correlation analyses of TRS with immune-related pathways. (B) The heatmap shows the relationship of TRS with immune-related pathways and clinicopathologic features. (C) Correlation analyses of TRS with tumor purity, stromal score, immune score, ESTIMATE score, relative infiltration abundance of multiple immunocytes, and immune checkpoints. (D) The heatmap shows the relationship of TRS with tumor purity, stromal score, immune score, ESTIMATE score, relative infiltration abundance of multiple immunocytes, immune checkpoints, and clinicopathologic features. (E) Correlation analyses of TRS with leukocyte fraction, lymphocyte infiltration signature score, TGF-beta response, and TIL regional fraction. *p < 0.05, **p < 0.01, and ***p < 0.001.

Given the critical importance of the TME in carcinogenesis, the relationship between TRS and the TME was explored. The correlation analyses showed that TRS was remarkably negatively associated with almost all immune-related pathways. The low TRS group showed more active immune-related pathways and better outcomes than the high TRS group (Figs. 6A and 6B). We also found that TRS had an opposite trend with respect to the ESTIMATE score, immune score, and stromal score. Meanwhile, it had a similar trend with respect to tumor purity. The individuals with high TRS were found to have a higher tumor purity with worse outcomes, while individuals with low TRS were observed to have higher ESTIMATE, immune, and stromal scores with better survival. Multiple immunocytes were also distributed differently between those with low and high TRS. The infiltration of T cells CD4 memory resting, dendritic cells resting, and B cells memory was particularly high in those with low TRS, while increased infiltration of immunosuppressive Tregs was observed in those with high TRS. In those with low TRS, most of the immune checkpoints were remarkably upregulated, with the exception of programmed cell death ligand 1 (PD-L1) (Figs. 6C and 6D). In addition, we observed a moderate and negative correlation of TRS with the lymphocyte infiltration signature score (Pearson coefficient R = −0.48, *p* < 2.2e-16) and a weak and inverse correlation of TRS with leukocyte fraction (Pearson coefficient R = −0.29, *p* = 1e-10). TRS did not correlate with TGF-beta response or TIL regional fraction (Fig. 6E). From these results, we concluded that a high TRS was associated with an immunosuppressive TME, whereas a low TRS was relevant to an immune-active TME.

### Genomic alterations in different TRS groups

**Figure 7:**
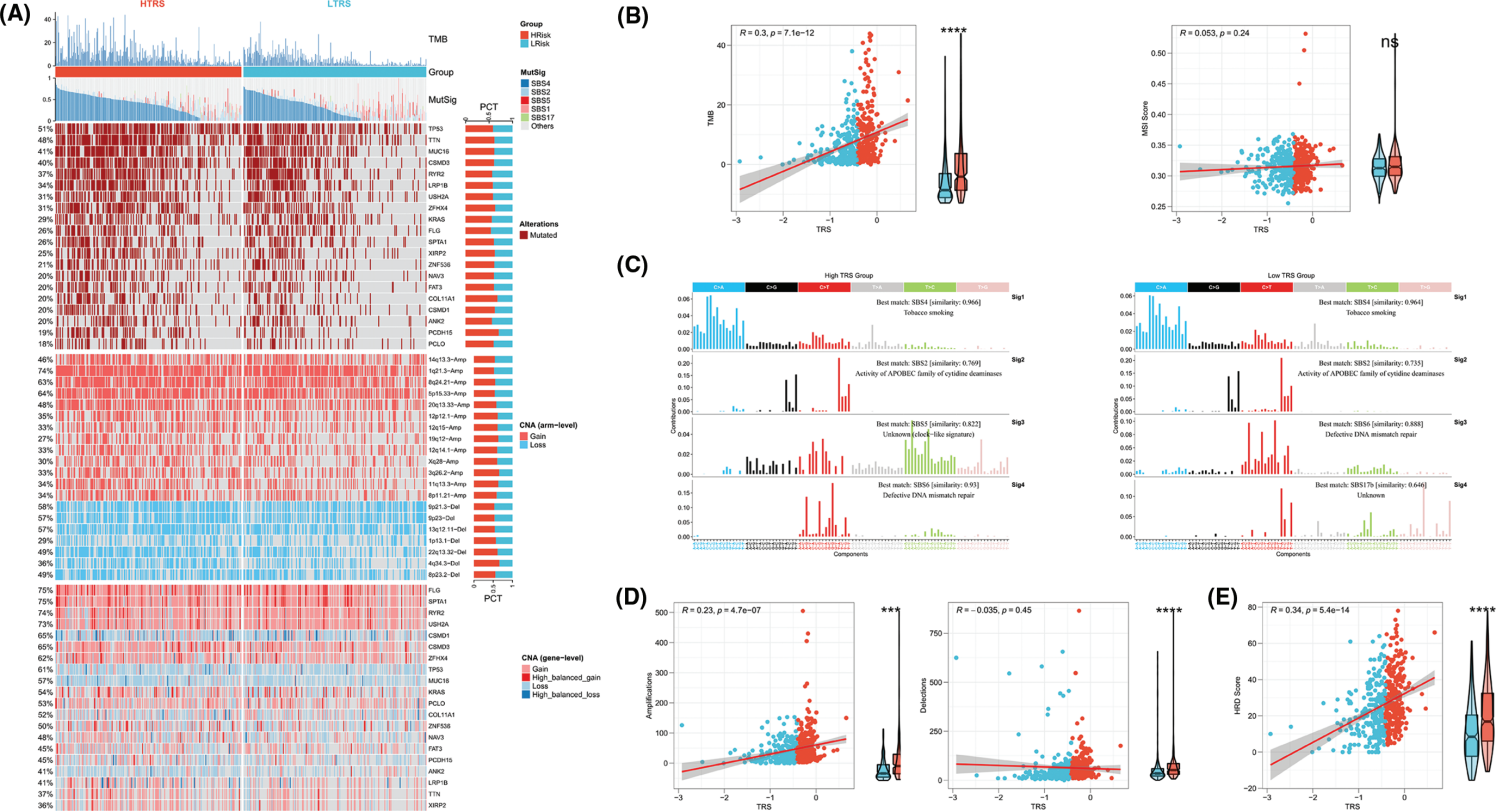
Genomic alterations in different TRS groups. (A) Heatmap showing TMB, mutational signature (MutSig), driver mutant genes, CNA (arm level), and CNA (gene level) in both subgroups. (B) Correlation analyses of TRS with TMB and MSI score. (C) The four mutation signatures were each detected in both groups. (D) Correlation analyses of TRS with amplifications and deletions of the chromosomal arm. (E) Correlation analyses of TRS with HRD score.

To further identify the possible mechanisms leading to differential tumor immunity between the different TRS groups, we examined the genomic alterations in the two subgroups. Fig. 7A summarizes the landscape of TMB, mutational signature, driver mutant genes, and CNAs in different TRS groups. Considering that TMB was a crucial indicator for representing mutation accumulation in cancer, we first examined TMB between these two subgroups. As shown in Fig. 7A, the TMB was much higher in those with high TRS than in those with low TRS. Consistent with this phenomenon, a positive correlation was observed for TRS and TMB in Fig. 7B (Pearson coefficient R = 0.3, *p* = 7.1e-12). MSI were hypermutated patterns of genomic microsatellites resulting from mismatch repair defects. Our findings showed that the TRS did not correlate with the MSI score (Pearson coefficient R = 0.053, *p* = 0.24; Fig. 7B). The mutational signature could decipher the complex somatic mutation patterns in the cancer genome. We examined the mutational signatures in the two subgroups and found that the SBS4 signature (associated with tobacco smoking) was predominant in both subgroups, suggesting that the same leading carcinogenic factors are present in both subgroups. Both the SBS2 signature (related to APOBEC family cytidine deaminase activity) and the SBS6 signature (related to impaired DNA mismatch repair) occurred in the two subgroups. The SBS5 signature occurred exclusively in the high TRS group, whereas the SBS17b signature was present only in the low TRS group (Figs. 7A and 7C). The 20 driver mutant gene frequencies were also analyzed in different TRS groups. The high TRS individuals had substantially higher mutation frequencies of 20 driver mutant genes than the low TRS individuals (Fig. 7A). CNAs are highly prevalent in cancer and have been found to contribute significantly to cancer progression. Fig. 7A also shows the CNAs at the chromosomal arm level and at the gene level in the two subgroups. The high TRS group showed increased CNAs, including gains and losses at the chromosomal arm level. We further examined the CNAs of 20 driver mutant genes in the two subgroups. Fig. 7A shows that the CNAs of 20 driver mutant genes increased significantly in the high TRS individuals. Correlation analyses showed a positive correlation between TRS and chromosomal arm amplifications (Pearson coefficient R = 0.23, *p* = 4.7e-07; Fig. 7D), whereas no correlation was found between TRS and chromosomal arm deletions (Pearson coefficient R = −0.035, *p* = 0.45; Fig. 7D). HRD is widely recognized as the driver producing CNAs. As expected, Fig. 7E revealed that the TRS was moderately and positively correlated with the HRD score (Pearson coefficient R = 0.34, *p* = 5.4e-14). Thus, the implication of these data was that the group with high TRS had more genomic alterations and was more susceptible to carcinogenesis.

### The role of TRS in predicting the benefits of chemotherapy

**Figure 8:**
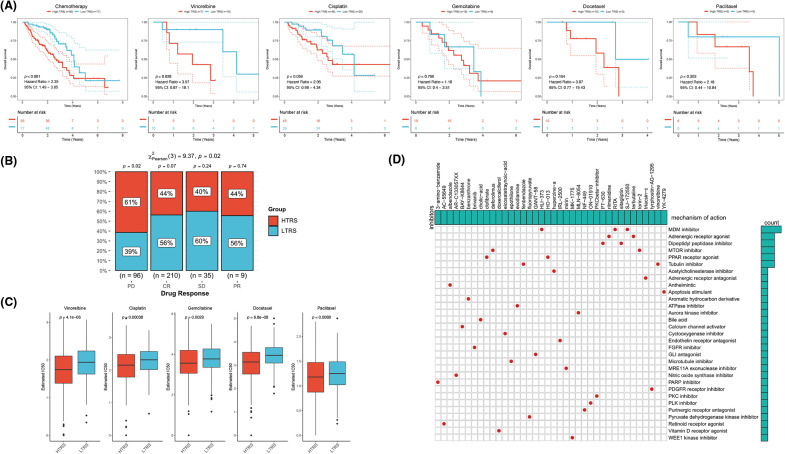
The role of TRS in predicting chemotherapy benefits. (A) Kaplan‒Meier curve for patients receiving chemotherapy, vinorelbine, cisplatin, gemcitabine, docetaxel, or paclitaxel. (B) Clinical response to chemotherapeutic agents in both subgroups. (C) The IC50 estimates of vinorelbine, cisplatin, gemcitabine, docetaxel, and paclitaxel in both subgroups. (D) The heatmap shows the small molecule drug candidates in the column and their common mechanism of action in the rows utilizing the CMap database.

**Figure S3:**
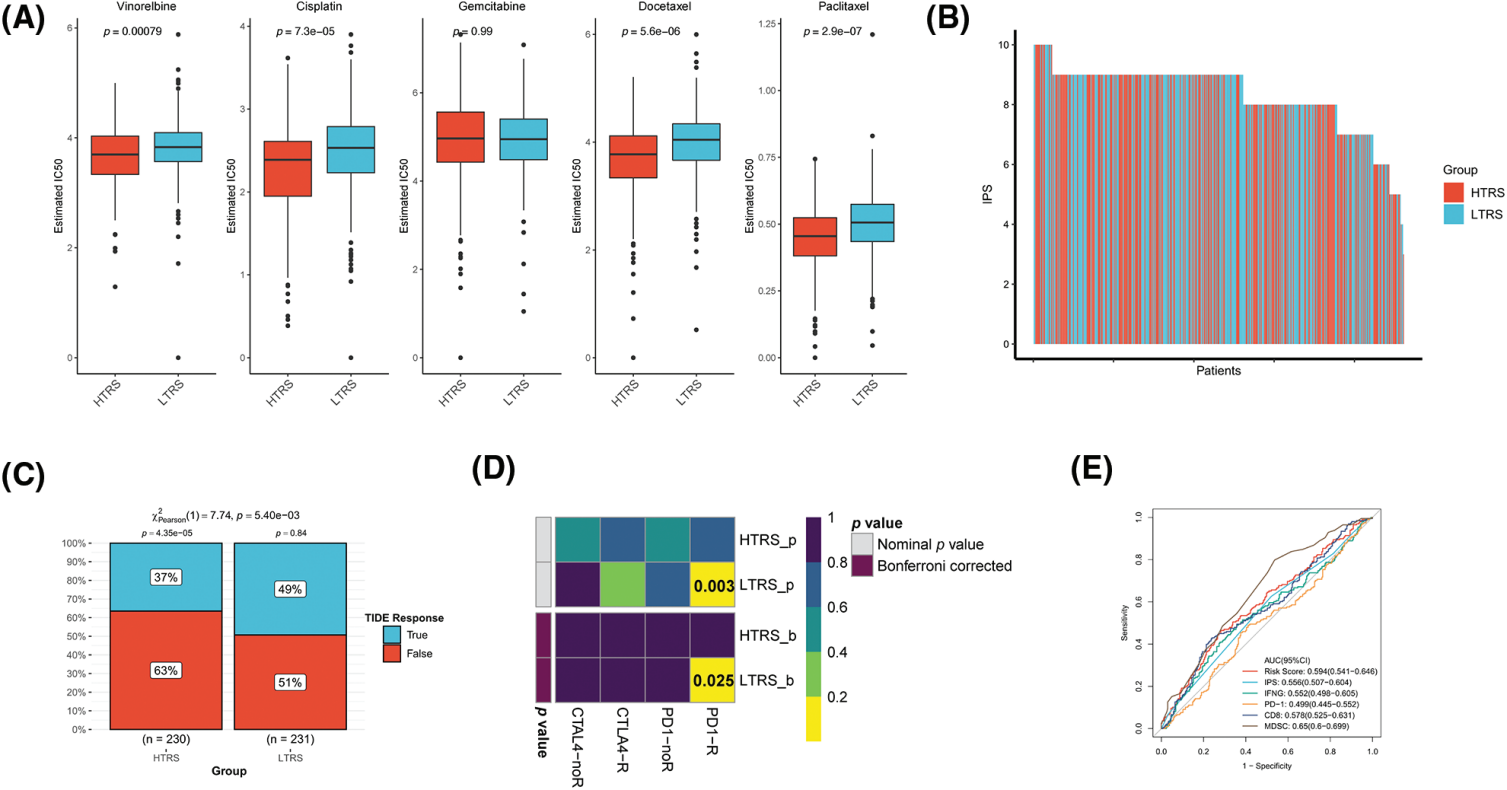
Prediction of therapeutic benefits based on TRS in GEO-LUAD validation cohort. (A) The IC50 estimates of vinorelbine, cisplatin, gemcitabine, docetaxel, and paclitaxel in both subgroups. (B) The distribution of IPS in the two subgroups. The TIDE algorithm (C) or subclass mapping algorithm (D) was performed to predict the anti-PD-1 and anti-CTLA4 immunotherapy responses for LUAD patients in both subgroups. (E) ROC curves of TRS and the other five biomarkers for predicting response to immunotherapy.

To date, chemotherapy has been an indispensable part of LUAD treatment. Therefore, our focus was to determine whether TRS could serve as a predictor of chemotherapy benefit. As shown in Fig. 8A, patients with different TRS treated with chemotherapy showed different survival rates. Those with low TRS showed improved outcomes compared with those with high TRS. When treated with vinorelbine, the results were significantly better in those with low TRS. For patients treated with cisplatin, gemcitabine, docetaxel, and paclitaxel, survival in the two subgroups was not significantly different, but those with high TRS exhibited a trend toward inferior outcomes relative to those with low TRS. We then examined the utility of TRS to predict the clinical response to chemotherapy. The rate of progressive disease (PD) differed statistically between the two subgroups. Those with high TRS had a PD rate as high as 61%, far exceeding the 38% PD rate of those with low TRS. In contrast, those with low TRS showed much higher rates of partial response (PR), stable disease (SD), and complete response (CR) relative to those with high TRS (Fig. 8B). These findings raised the possibility that the difference in response to chemotherapy may be partly responsible for the different survival of the two subgroups. In addition, whether different TRS contributed to the differences in sensitivity to chemotherapeutic agents was investigated. Both subgroups had significantly different sensitivities to paclitaxel docetaxel, vinorelbine, gemcitabine, and cisplatin, as indicated by the IC50 estimates. Those with high TRS had lower IC50 estimates for all five chemotherapeutic agents, indicating that those with high TRS could be more sensitive to all five agents (Fig. 8C). In addition, high TRS individuals from the GEO cohort also exhibited significantly lower IC50 estimates for all five agents (Suppl. Fig. S3A). To explore potential agents for the treatment of LUAD, 150 DEGs with the highest degree of upregulation or downregulation between the two subgroups were entered into the CMap database. A set of 38 small molecule drug candidates were screened, and a set of 30 modes of interaction were uncovered (Fig. 8D). Simply put, these findings underscore the significant value of TRS in predicting chemotherapy benefit and antitumor drug selection for LUAD patients.

### Evaluation of the immunotherapy response based on TRS

**Figure 9:**
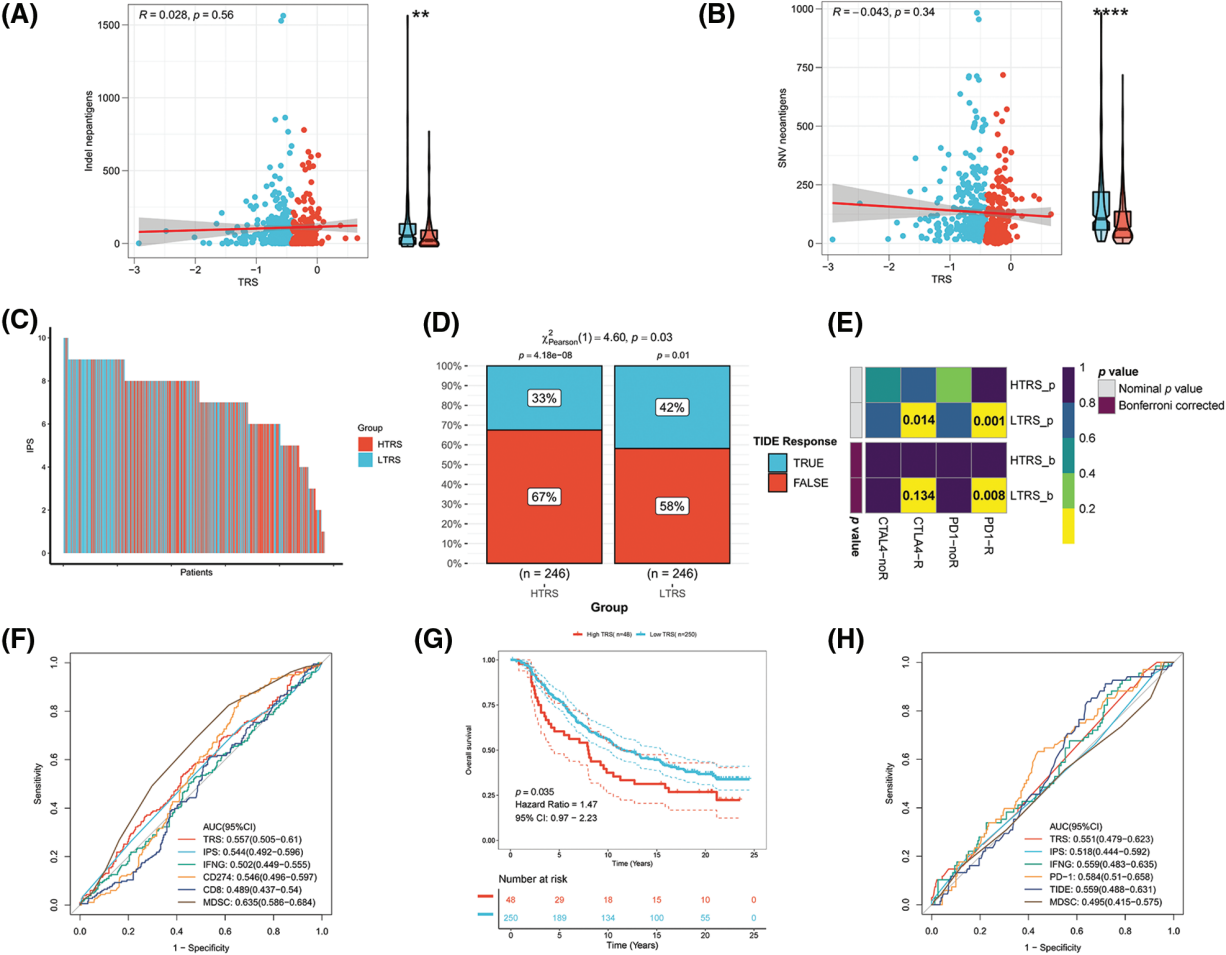
Evaluation of the immunotherapy response based on TRS. Correlation analyses of TRS with Indel neoantigens (A) and SNV neoantigens (B). (C) The distribution of IPS in the two subgroups. The TIDE algorithm (D) or subclass mapping algorithm (E) was performed to predict the anti-PD-1 and anti-CTLA4 immunotherapy responses for LUAD patients in both subgroups. (F) ROC curves of TRS and the other five biomarkers for predicting response to immunotherapy in the TCGA-LUAD training cohort. (G) Kaplan–Meier curve for patients in the two subgroups from the IMvigor210 cohort. (H) ROC curves of TRS and the other five biomarkers for predicting response to immunotherapy in the IMvigor210 cohort.

Immunotherapy, especially immune checkpoint blockade (ICB), has experienced major advances in the past few years, which has significantly changed the landscape of cancer treatment. Unfortunately, identifying predictive markers is an ongoing research area, as the bulk of patients are not responsive to immunotherapy [39]. Thus, we were further concerned about the availability of TRS in predicting the response to immunotherapy. Tumor neoantigens are of great importance for the immunotherapy of cancer. In particular, Indel and SNV derived tumor neoantigens are the most extensively investigated classes of tumor neoantigens [40]. Figs. 9A and 9B show no correlation of TRS with Indel neoantigens (Pearson coefficient R = 0.028, *p* = 0.56) or SNV neoantigens (Pearson coefficient R = −0.043, *p* = 0.34). Nevertheless, the low TRS group tended to have more neoantigens than the high TRS group. In addition to being a prognostic biomarker, the IPS was also found to be a superior biomarker for the prediction of response to immunotherapy with PD-1 blockers and CTLA-4 blockers. Following the preceding study, the IPS of LUAD patients with distinct TRS was determined, and a higher IPS indicated a better immunotherapy response. As shown in Fig. 9C, the patients with low TRS demonstrated markedly increased IPS in contrast to the patients with high TRS, implying that those with low TRS could respond better to checkpoint blockers. At the same time, the responses to anti-PD-1 and anti-CTLA-4 treatments in the two subgroups were predicted utilizing the TIDE algorithm. The rate of those who responded to ICB therapy was considerably higher in patients with low TRS than in patients with high TRS, as shown in Fig. 9D (*p* = 0.03). Taking advantage of the 47 melanoma cases already reported with detailed information on immunotherapy, a subclass mapping algorithm was also performed to visualize treatment response. The heatmap showed that those with low TRS responded better to treatment with anti-PD-1 than those with high TRS (Bonferroni corrected *p* = 0.008; Fig. 9E). The GEO-LUAD validation cohort also reported similar findings (Suppl. Figs. S3B–S3D). We further evaluated the accuracy of the TRS in the prediction of response to immunotherapy with the use of the ROC curve. The findings revealed that the predictive power of TRS was inferior to that of the myeloid-derived suppressor cell (MDSC), but better than that of the other four biomarkers for immunotherapy in the TCGA-LUAD training cohort, including IPS, interferon gamma (IFNG), CD274, and CD8 (Fig. 9F). We also compared the predictive power of TRS and five other immunotherapy biomarkers, including IPS, IFNG, PD-1, CD8, and MDSC in the GEO-LUAD cohort (Suppl. Fig. S3E). In addition, patients in the IMvigor 210 cohort were categorized as high/low TRS based on median TRS. In accordance with the abovementioned findings, those with high TRS from the IMvigor210 cohort experienced unfavorable survival (Fig. 9G). TRS in the IMvigor210 cohort could afford accuracy in predicting immunotherapy response, although TRS did not perform best compared with the other five immunotherapy indicators, including IPS, IFNG, PD-1, TIDE, and MDSC (Fig. 9H). Overall, these results suggested that TRS may have broad applications as a biomarker for predicting immunotherapy benefits in LUAD individuals.

### Predictive efficacy of TRS from a pancancer perspective

**Figure 10:**
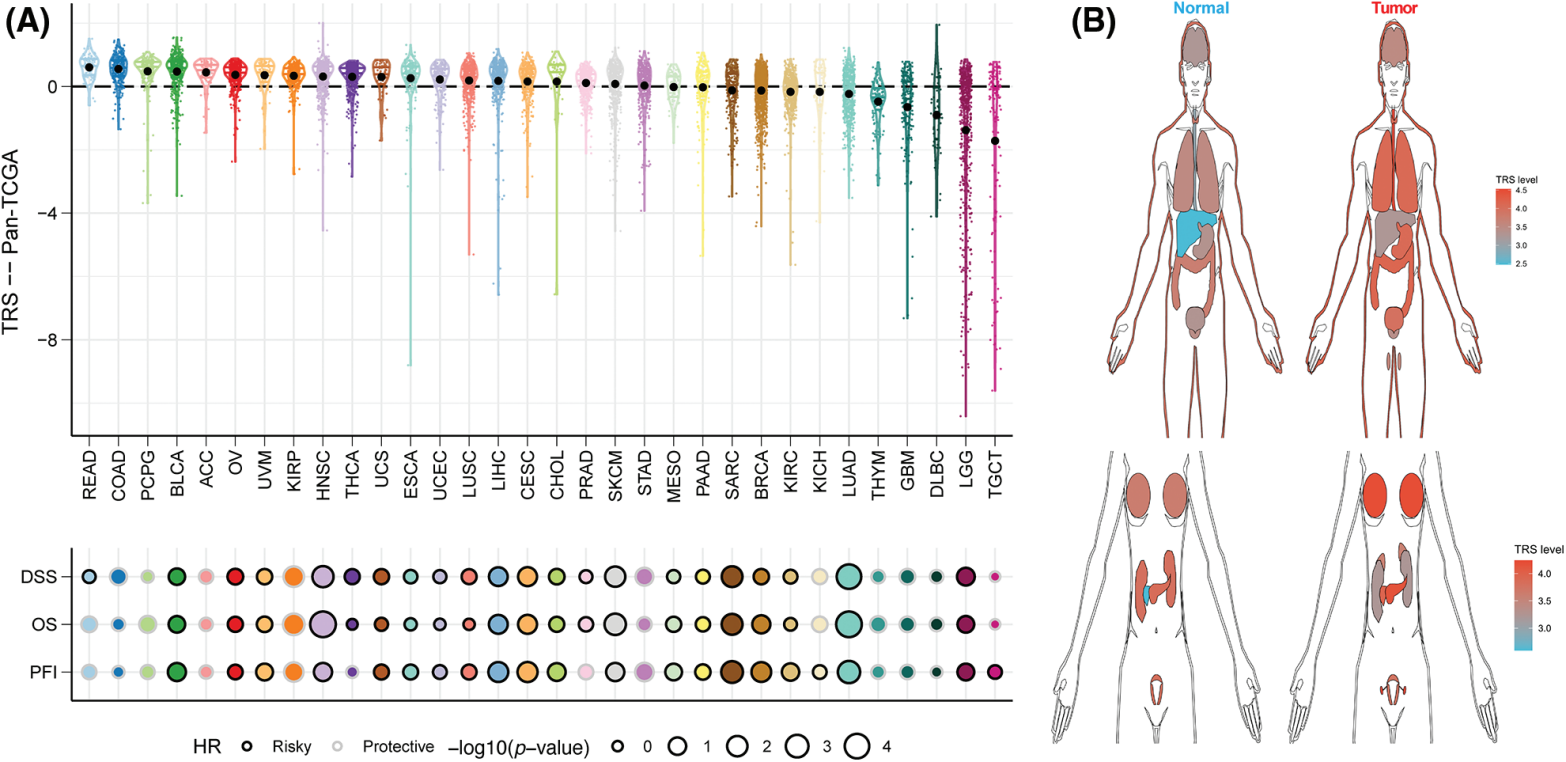
Predictive accuracy of the TRS model in the TCGA-pancancer cohort. (A) Distribution and predictive value of TRS in solid tumors in the TCGA pancancer cohort. (B) Differences in the distribution of TRS in normal and tumor tissues in different organs.

To assess the generalizability of TRS application in different solid tumors, we constructed TRS in the TCGA-pancancer cohort and evaluated the distribution and predictive efficacy of TRS. Our results showed a significant distribution of TRS in most solid tumors, with the highest evaluated TRS being in colorectal and bladder cancers (Fig. 10A). In addition, TRS can be a significant risk factor for bladder cancer, head and neck cancer, liver cancer, melanoma and soft tissue sarcoma (Fig. 10A). Finally, we evaluated the differential expression of TRS in normal and tumor tissues in different organs. The results showed that TRS was significantly elevated in tumor tissue in most organs of the human body except the kidney (Fig. 10B).

### The impact of LINC00525 on LUAD cell proliferation or invasion

**Figure 11:**
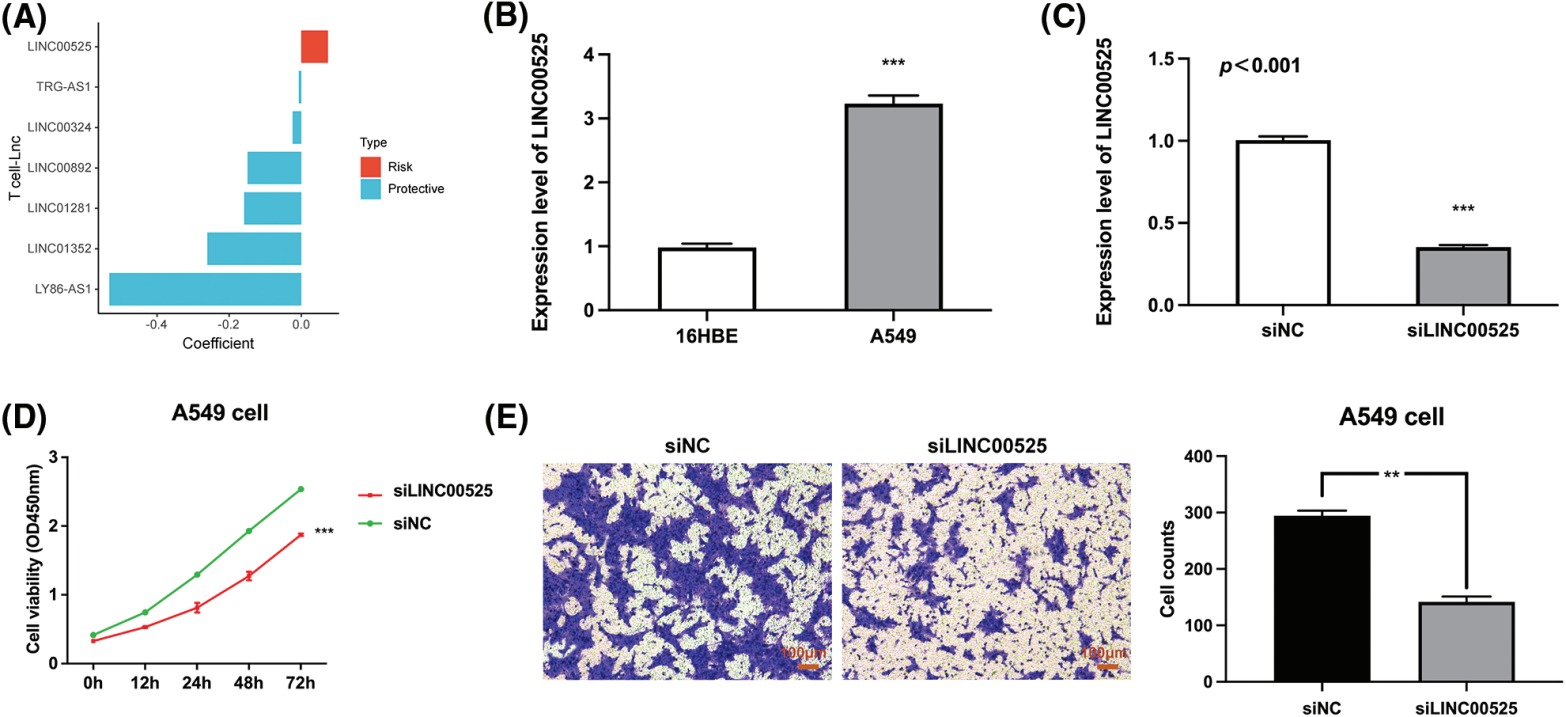
The impact of LINC00525 on LUAD cell proliferation or invasion. (A) The risk coefficients of seven T-cell-Lncs. (B) LINC00525 expression in 16HBE cells and A549 cells (n = 3). (C) LINC00525 expression in A549 cells transfected with siNC or siLINC00525 (n = 3). (D) The viability of A549 cells transfected with siNC or siLINC00525 (n = 3). (E) The invasion of A549 cells transfected with siNC or siLINC00525 (n = 3). Scale bar = 100 μm. *p < 0.05, **p < 0.01, and ***p < 0.001.

Finally, we analyzed the risk coefficients of seven T cell-Lncs in the prognostic model. According to Fig. 11A, the risk coefficient of LINC00525 was the highest among the seven T cell-Lncs. In the prognostic model, the other six T cell-Lncs served as protective factors, while only LINC00525 appeared to be a risk factor. Therefore, LINC00525 was selected as a functional study target. LINC00525 expression changes were initially examined in the normal bronchial epithelial cell strain and the LUAD cell strain. In contrast to 16HBE cells, LINC00525 expression was dramatically elevated in A549 cells (Fig. 11B). However, LINC00525 expression was remarkably downregulated in siLINC00525-transfected A549 cells relative to siNC-transfected A549 cells (Fig. 11C). The impact of LINC00525 on proliferating A549 cells was determined by the CCK8 assay. Compared to A549 cells transfected with siNC, A549 cells transfected with siLINC00525 demonstrated a striking decrease in cell viability (Fig. 11D). LINC00525 was also evaluated for its ability to induce A549 cell invasion. The ability of A549 cells transfected with siLINC00525 to invade was apparently impaired, in contrast to that of A549 cells transfected with siNC (Fig. 11E). Taken together, these findings implicated LINC00525 in helping LUAD cells proliferate and invade.

## Discussion

The success of cancer immunotherapy in treating LUAD has been consistently demonstrated. However, the efficacy of treatment is variable and dependent partly upon the number and characteristics of TILs [41]. Among multiple populations of tumor-invading immunocytes, such as lymphocytes, macrophages, mast cells, and dendritic cells, TILs have been identified as a select T cell population with higher immunologic specificity toward tumor cells relative to non-infiltrating lymphocytes [6]. For example, it has been recognized for more than 20 years that in patients with melanoma, a robust influx of T cells is linked to improved OS [42]. However, the blanket assumption that T cell infiltration of cancer lesions is always favorable for the patient may be inappropriate. In both solid and hematologic tumors, the effector/regulatory T cell ratio (Teff/Treg) has been proven to be associated with OS [43]. Furthermore, T-cell activity is critical in the matrix and is linked to survival in nearly all types of tumors [44]. LncRNAs are a new subset of noncoding RNAs and are diffusely expressed in human cells. LncRNAs are regulators of a variety of biological processes through different mechanisms [45]. A growing body of research has found that lncRNAs participate in both natural and acquired immune defenses by modulating the differentiation and function of immunocytes [46]. In particular, virtually all cancers currently have aberrant lncRNA profiles. New research has identified some lncRNAs dysregulated in LUAD that are related to the progression and outcome of carcinoma [47,48]. These findings implicate lncRNAs as promising diagnostic tools and prognostic predictors in LUAD. However, T cell-Lncs have rarely been reported as a prognostic signature in patients with LUAD. Therefore, we aimed to identify lncRNAs that correlate with various T cell subsets in LUAD and develop TRS to better stratify LUAD patients.

In the first step, we identified 16 T cell-Lncs as candidates in this study using the two algorithms (ImmLnc algorithm and WGCNA algorithm). Then, through univariate Cox analysis, a total of 10 T cell-Lncs with significance in prognosis were filtered out. Finally, the most robust TRS model was created using the LASSO algorithm. The prognostic model performed best when seven T cell-Lncs (LINC00324, LINC00892, LINC01281, LINC01352, LY86-AS1, TRG-AS1, and LINC00525) were included. We calculated TRS for each patient, taking into account heterogeneity between patients. Patients with low TRS were found to survive longer in both the TCGA-LUAD training cohort and the GEO-LUAD validation cohort, implying that TRS may function as an adverse prognostic biomarker. Analysis of ROCs confirmed the excellent predictive power of TRS for survival at 1, 3, and 5 years in both the training TCGA-LUAD cohort and the validation GEO-LUAD cohort. Compared with age, gender, and stage, TRS had superior OS prediction in each cohort. Further Cox regression analyses revealed TRS as an independent and robust predictor of OS for both the TCGA-LUAD cohort and GEO-LUAD cohort. Our data also demonstrated the good predictive power of the TRS-based nomogram, which could serve as a statistical tool with large clinical applications to evaluate the overall probability of a particular outcome in individual LUAD patients. Next, we examined the molecular characteristics of LUAD patients with different TRS. The LUAD patients with high TRS showed more active cell proliferation, while LUAD patients with low TRS showed a more active immune landscape. Those with high TRS carried substantially more genomic mutations than those with low TRS. These results were largely responsible for the worse outcome of LUAD patients with high TRS. We also wanted to see how TRS relates to TME. High TRS was associated with an immunosuppressive TME, whereas low TRS was relevant to an immune-active TME. It was assumed that TRS can characterize and represent the TME to some degree. In addition, we further investigated the utility of TRS for clinical management. High TRS individuals were quite susceptible to chemotherapy, while low TRS individuals were more responsive to immunotherapy. The good accuracy of TRS in predicting immunotherapy response was also validated in the GEO and IMvigor210 cohorts. These results therefore confirm the substantial value of the TRS both as an indicator of outcome and as a biomarker for the prediction of treatment response.

The application of chemotherapeutic agents for NSCLC is longstanding. Palliative chemotherapy continues to be the cornerstone of management in patients with advanced NSCLC [4]. Patients who are doing well are now receiving first-line platinum-based chemotherapy in combination with any of the following cytotoxic agents: gemcitabine, vinorelbine, paclitaxel, pemetrexed, docetaxel, or nab-paclitaxel [49]. It relieves tumor-related symptoms and improves overall patient survival [50]. However, there is evidence that chemotherapy can stimulate immunosuppressive cells to form in the TME of various malignancies [51]. In human pancreatic cancer, chemotherapy has been shown to induce monocytes to differentiate into MDSCs in the TME [52]. More recently, cancer chemotherapeutic drugs have been shown to alter the TME, and these alterations may in turn affect the ultimate efficacy of treatment [53,54]. Increased infiltration of macrophages has been noted in breast cancer patients after chemotherapy. These macrophages may in turn protect against tumor cell death, which is triggered by a number of different chemotherapeutic drugs [55]. In the present study, high TRS individuals had relatively elevated sensitivity to the five chemotherapy drugs vinorelbine, gemcitabine, cisplatin, paclitaxel, and docetaxel. However, individuals with high TRS showed an unfavorable clinical course and lower chemotherapeutic response. It is possible that these therapeutic interventions profoundly altered the TME and that these alterations, in turn, critically attenuated the response to chemotherapy. Thus, a thorough understanding of how cytotoxic therapy interacts with the TME is needed to help improve the treatment of LUAD.

In addition, in-depth comprehension of tumor immunity is essential for advancing immune-based therapeutic strategies [56]. Advances in immunotherapy have enabled a subset of LUAD patients to achieve durable, long-term successes [57]. Understanding the subgroups of patients who respond to ICB therapy at the cellular level and in the context of the TME is becoming increasingly important [58]. The TME, which contains tumors and immune cells, is a heterogeneous milieu. The immune landscape of the TME can restrain or boost tumorigenesis and progression [59]. Thus, there is an urgent need for biomarkers that can better depict the TME and predict outcomes to identify responders/non-responders. In this study, LUAD patients with high and low TRS had completely different tumor immune status in their TME. The low TRS individuals were marked by an active immune status, increased stromal and immune scores, and decreased tumor purity compared with high TRS individuals. Recently, several studies have shown that patients with an active immune signaling profile are candidates for immunotherapy in various solid tumor types [56]. Consistent with these results, individuals with low TRS had significantly higher odds of responding to immunotherapy. These results suggest that the TRS determined in this study may characterize the TME and be a better predictor of immunotherapy efficacy.

In the present work, we identified seven T cell-Lncs to create TRS for LUAD patients. While the six T cell-Lncs, including LINC00324, LINC00892, LINC01281, LINC01352, LY86-AS1, and TRG-AS1, were protective factors, LINC00525 was a risk factor. LINC00892 demonstrated exclusive expression in T cells that have been differentiated. In particular, CD4^+^ effector memory subtypes exhibit the highest expression of LINC00892. This enables LINC00892 to be a potential biomarker for activated T cells [60]. LINC01352 is notably downregulated in hepatitis B virus (HBV)/HBV X protein (HBx)-positive liver cancer cells and tissues. It might be responsible for HBx-induced tumor progression as a tumor-suppressing gene [61]. LINC00525 is a novel lncRNA with oncogenic activity in colon cancer. LINC00525 expression is remarkably elevated in colon cancer cells and tissues, and LINC00525 overexpression has been linked to adverse prognosis in colon carcinoma [62]. However, LINC00525 is still under description and characterization. Because LINC00525 was the only risk factor in the prognostic model, we identified LINC00525 as a valuable study target. qRTPCR analysis revealed overexpression of LINC0052 in LUAD cells. CCK-8 and transwell assays demonstrated that LINC00525 can help LUAD cells proliferate and invade. These results point to LINC00525 as a promising target for the treatment of LUAD.

Apart from the encouraging results of this work, we were also aware of the limitations of the present study. Although our research has the merit of using large cohorts from the TCGA and GEO databases for the generation and verification of the TRS prognostic model, the present study is still retrospective in nature. There is a need for a prospective cohort study to further validate the utility of the TRS. Additionally, we built the TRS prediction model using public datasets. The predictive power needs further verification in randomized controlled trials.

## Conclusion

In the present work, we identified seven T cell-Lncs to create the most robust prognostic model TRS. LUAD patients were classified into high/low groups using the median TRS. The two subgroups showed heterogeneity in clinical outcomes, genomic alterations, and the immune landscape of their TME. In addition, the TRS was able to accurately predict the outcome and response to chemotherapy or immunotherapy in patients with LUAD. LINC00525, as the only risk factor in the prognostic model, could promote LUAD cell proliferation and invasion. Overall, the TRS established by T cell-Lncs could unambiguously classify LUAD patients, predict their prognosis and guide their management. Moreover, our identified T cell-Lncs could provide potential therapeutic targets for LUAD.

## Supplementary Materials

Table S1 The gene signature for selected pathways.

Table S2 The risk coefficients of seven T cell-Lncs.

## Data Availability

The original datasets in this study were obtained from public online databases. Details can be found in the article/supplementary materials.
